# Do Non-Functional Adrenal Adenomas Affect Metabolic Profile and Carotid Intima-Media Thickness? A Single Centre Study from Poland

**DOI:** 10.3390/jcm12144612

**Published:** 2023-07-11

**Authors:** Magdalena Szychlińska, Magdalena Rzeczkowska, Katarzyna Gontarz-Nowak, Wojciech Matuszewski, Elżbieta Bandurska-Stankiewicz

**Affiliations:** 1 Clinic of Endocrinology, Diabetology and Internal Medicine, Department of Internal Medicine, School of Medicine, Collegium Medicum, University of Warmia and Mazury, 10-561 Olsztyn, Poland; katarzyna.gontarz.92@gmail.com (K.G.-N.); wmatuszewski82@wp.pl (W.M.); bandurska.endo@gmail.com (E.B.-S.); 2 Department of Imaging, Provincial Specialist Hospital in Olsztyn, 10-561 Olsztyn, Poland; slowinska1@o2.pl

**Keywords:** adrenal incidentaloma, carotid-intima media thickness, metabolic

## Abstract

**Background:** Compared to the general population, among people with adrenal incidentalomas (AIs) the diagnosis of obesity, hypertension, impaired carbohydrate and lipid metabolism is more common. The aformentioned disorders represent typical cardiovascular remodeling risk factors. The study was designed to assess the association between NFAIs, metabolic profile and carotid intima-media thickness (CIMT) as the predictive factor of atherosclerosis. **Material:** The study included 48 patients with NFAI (16 men, 32 women, mean age 58.6 +/− 9 years) and 44 control participants (15 men, 29 women, mean age 57 +/− 7 years). Both groups were matched for age, gender and BMI. Subjects with history of myocardial infarction, stroke or diabetes mellitus (DM) were excluded. Participants underwent adrenal imaging, biochemical evaluation, and measurement of CIMT. Hormonal evaluation was conducted in AI patients. **Results:** The NFAI group had significantly higher waist circumference (*p* < 0.01), higher systolic (*p* < 0.01) and diastolic blood pressure (*p* < 0.01), fasting insulin (*p* = 0.03) and glucose in the 2 h OGTT (*p* = 0.04) as well as higher CIMT (*p* < 0.01). Hypertension (*p* < 0.01) and IGT (*p* = 0.026) were more common in this group as well. There was a positive correlation between CIMT and cortisol levels in 1 mg dexamethasone suppression test (r = 0.33, *p* = 0.02). **Conclusions:** Patients diagnosed with NFAIs, despite normal cortisol inhibition in the 1 mg dexamethasone test, still presented a number of metabolic abnormalities. The assessment of IMT may proove valuable in indicate the presence of early vascular remodelling in asymptomatic patients. The underlying mechanisms of these findings are still unknown, hence further studies are required.

## 1. Introduction

Adrenal incidentalomas (AIs) are common, asymptomatic lesions detected in imaging studies performed for reasons other than suspected adrenal pathology. The prevalence of AIs is estimated around 3–10% in general population. By definition, patients with AIs do not present clinical features of excess of adrenal hormones. Nearly 80% of adrenal lesions are benign, hormonally inactive adenomas (non-functional adrenal incidentalomas, NFAI). The most common endocrine disorder found in AI patients is excessive cortisol secretion, with a prevalence of 1% to 29%. The recommended diagnostic test for hypercortisolism is 1 mg overnight dexamethasone suppression test: serum cortisol <1.8 µg/dL excludes hypercortisolism, 1.9–5 µg/dL suggests possible autonomic cortisol secretion, and >5 µg/dL suggests autonomous cortisol secretion (ACS). It is suggested that cortisol secretion is a continuum between normal serum cortisol concentration and overt hypercortisolism and shows significant variability in one patient. The median prevalence of primary hiperaldosteronism is 2.5%, while pheochromocytma is estimated around 7%. Only adrenal tumors with clinically overt hormone excess are an indication for adrenalectomy [1,2,3,4,5,6].

The presence of AI is known to be associated with higher prevalence of cardiovascular risk factors, such as obesity, hypertension, carbohydrate and lipid metabolism disorders- namely conditions closely related to insulin resistance (IR) [7,8,9].

The pathogenetic mechanisms responsible for the development of AIs still remain unknown. A possihle link between AIs and hyperinsulinemia and the insulin-like growth factor (IGF) system has been suggested in the literature. IR and compensatory hyperinsulinemia through activation of insulin and IGF-1 receptors may play important role in adrenal tumor growth [10]. It remains unclear whether adrenal lesions develop as a result of primary IR and compensatory hyperinsulinemia, or whether IR is merely secondary to a slightly excessive cortisol secretion caused by AIs.

Interestingly, patients with clinically silent NFAI, with normal inhibition of cortisol secretion in the low-dose dexamethasone suppression test (LDDST), more frequently present with metabolic disorders, including the development of metabolic syndrome (MS) as well as morphological and functional changes in the endothelium. Seemingly asymptomatic patients with NFAI are diagnosed with early stages of cardiovascular remodelling [11,12,13].

The study was designed to assess the impact of NFAIs on the metabolic profile and early stages of atherosclerosis in the carotid arteries.

## 2. Subjects and Methods

### 2.1. Ethics and Setting

This clinical study was conducted in the Clinic of Endocrinology, Diabetology and Internal Medicine at the Regional Specialist Hospital in Olsztyn between January 2020 and December 2020. From total of patients with AI hospitalized in our department patients with non-secreting AI were selected. The study was approved by the Bioethics Committee of the Warmia-Mazury Medical Chamber. Written informed consent was obtained from all recruited subjects.

### 2.2. Subjects

The study included 48 patients with AI. Radiological, biochemical and hormonal diagnostics were performed as part of the standard procedure for patients with AI in accordance with the guidelines of the European Endocrine Society [3,4,5]. In addition, patients gave written informed consent to CIMT measurement with non-invasive ultrasound method. The control group, examined between January 2020 and December 2020, consisted of 44 asymptomatic volunteers age-, gender-, *ethnicity*- and BMI-matched. Written informed consent to blood examination, abdominal ultrasonography and CIMT measurement was collected from each patient. Subjects with history of myocardial infarction, stroke or diabetes mellitus (DM) were excluded. Exclusion criteria are listed in Table 1.

### 2.3. Study Protocol

#### 2.3.1. Medical Interview and Physical Examination

In both groups, an interview and a physical examination with anthropometric measurements (weight, height, waist circumference) were performed. Blood pressure (BP) was measured twice from the upper right arm after 5 min of rest in a seated position, and the mean of these two measurements was taken as the BP value. Hypertension was diagnosed on the basis of two criteria: systolic blood pressure ≥ 140 mmHg and diastolic blood pressure ≥ 90 mmHg or, in the case of treatment with antihypertensive drugs, according to the guidelines of the European Society of Cardiology (ESC) and the European Society of Hypertension (ESH) [14,15].

#### 2.3.2. Biochemical Evaluation

Blood samples for baseline laboratory tests were collected from the ulnar vein in the supine position, in the morning, after 12 h of fasting. Laboratory tests included measurements of sodium, potassium, creatinine, uric acid, fasting glucose and insulin, lipid profile, 75 g glucose load test (OGTT). The insulin resistance index was calculated according to the Homeostasis Model Assesment-Insulin Resistance (HOMA-IR) formula from the following equation (HOMA-IR = (fasting plasma glucose concentration × fasting plasma insulin concentration)/22.5). HOMA-IR < 2.5 was taken as the normal result [16]. Non-HDL concentration was calculated using the following formula (non-HDL-C = TC - HDL-C).

Dyslipidaemia was diagnosed on the basis of LDL cholesterol level (LDL-C) > 115 mg/dL, and/or total cholesterol (TC) > 150 mg/dL, and/or HDL cholesterol (HDL-C) < 40 mg/dL (men) and 50 mg/dL (women), or the use of lipid-lowering drugs according to the ESC and ESH guidelines [15].

Diabetes, impaired fasting glucose (IFG) and impaired glucose tolerance (IGT) were diagnosed according to American Diabetes Association guidelines [17].

According IDF consensus, MS was diagnosed in the presence of any three of the following: waist circumference greater than 94 cm in men and greater than 80 cm in women, triglycerides of 150 mg/dL (1.7 mmol/L) or more, HDL cholesterol less than 40 mg/dL (1.0 mmol/L) in men and less than 50 mg/dL (1.3 mmol/L) in women, blood pressure of 130/85 mm Hg or higher, or fasting glucose of 100 mg/dL or higher [18].

#### 2.3.3. Hormonal Evaluation

Normal hormonal activity of adrenal tumors in the study group was confirmed on the basis of normal diurnal cortisol rhythm (8 a.m., 10 p.m.), adrenocorticotropic hormone (ACTH) levels in the morning >5 pg/mL, cortisol levels below 1.8 ug/dL in a 1 mg, low dose dexamethasone suppression test (LDDST), normal adrenal androgen levels (dehydroepiandrosterone sulfate (DHEA-S), androstendione) After withdrawal of diuretics, beta-blockers, angiotensin-converting enzyme inhibitors, and angiotensin II receptor type 1 antagonists) plasma aldosterone concentration (PAC) and plasma renin activity (PRA) were measured. Aldosterone-renin ratio (ARR) was calculated as PAC (ng/dL) divided by PRA (ng/mL/h). ARR below 30 ng/dL/mL/h excluded hyperaldosteronism. Pheochromocytoma was excluded by normal limits of urinary fractionated metanephrines or normal plasma catecholamine levels [2,4,6]. Due to no changes in the adrenal glands hormonal evaluation of the control group was not performed.

#### 2.3.4. Abdominal Imaging Examination

**Figure 1:**
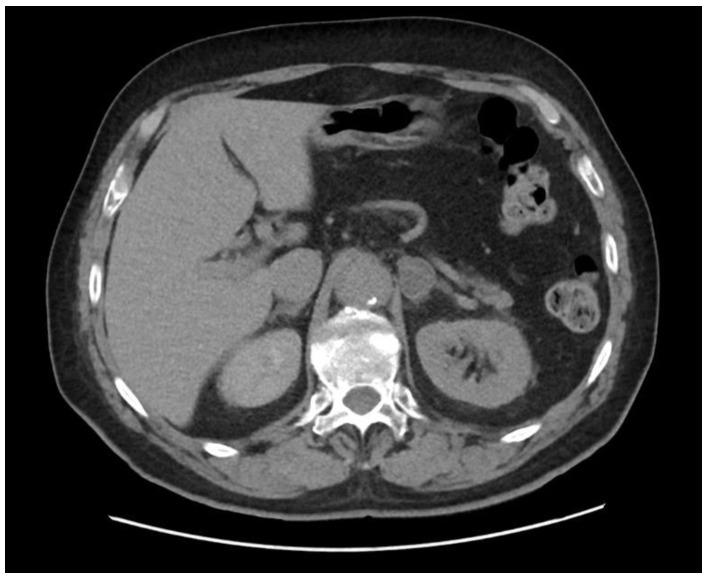
Non-contrast CT image showing AI.

AI was confirmed as a gold standard by computed tomography (CT). On CT scans, adrenal tumours in their largest dimension did not exceed 4 cm, they were regularly shaped with well-defined margins, homogeneous density, <10 HU in unenhanced CT, ≤30 HU after contrast (Figure 1). If adenomas were not possible to be diagnosed with CT, MRI was performed [2,4,6].

In the control group the presence of an adrenal tumour was excluded by abdominal ultrasound with a Samsung SAR7-EXP-CW. This imaging method was chosen due to no ionising radiation and relatively low price. In a large sample study the sensitivity, specificity, and accuracy of ultrasound-based diagnosis were 89%, 99%, and 93.9%, respectively [19].

#### 2.3.5. Carotid-Intima Media Tickness

**Figure 2:**
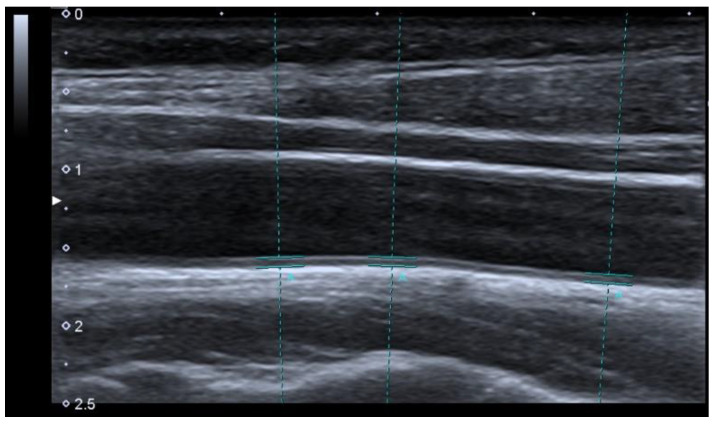
Intima-media thickness *ultrasound measurement* (A).

An experienced radiologist measured the carotid intima-media thickness (CIMT) in the supine position with the head tilted posteriorly using a Samsung SAR7-EXP-CW ultrasound scanner. In order to increase the repeatability of measurements, the examination was performed by the same specialist. Ultrasonography images were obtained of the right and the left common carotid artery of each patient at the lower 1/3 cervical region proximally and 1 cm above the carotid bulb distally in the longitudinal plane (Figure 2). CIMT values were calculated with the use of the arithmetic mean formula of the measured values [20].

### 2.4. Statistical Analysis

Statistica 13.0 PL software for Windows was employed for the statistical purposes. The mean and standard deviation (mean ± SD) were calculated for all the analysed parameters. Qualitative data were presented as indices of structure (%). The normality of distribution of the obtained results was calculated with the Shapiro-Wilk test. Student’s *t*-test was used to assess the statistical significance of differences between the analysed groups. If the variables did not meet the normality criteria, the Mann-Whitney U test was performed. Chi-square test were used to determine differences for categorical variables. Spearman’s rank correlation coefficient was applied to assess correlations between parameters. The significance level of all the performed statistical analyses was set at α = 0.05.

## 3. Results

The study encompassed a total of 48 patients with NFAI aged 58.6 +/− 9 years, 16 men (33%), 32 women (67%). The control group consisted of 44 people aged 57 +/− 7 years, 15 men (34%), 29 women (66%). The NFAI group showed a statistically significantly higher waist circumference, as well as systolic and diastolic blood pressure (Table 2).

As shown in Table 3, the NFAI group and the control group showed no significant differences in terms of fasting glucose level, HOMA-IR index, TC, LDL-C, HDL-C, non-HDL-C, TG and creatinine levels. The NFAI group showed statistically significantly higher concentrations of sodium (*p* = 0.02) and glucose in 2 h OGTT (*p* = 0.04) and higher CIMT (*p* < 0.01). In NFAI group mean CIMT value was 0.694 ± 0.158 for men and 0.619 ± 0.176 for women. In control group mean CIMT value was 0.516 ± 0.099 for men and 0.452 ± 0.117 for women. The differences in CIMT thickness by gender in both groups were not statistically significant.

As shown in Table 4, hypertension (X^2^ = 7.89, *p* < 0.01) and IGT (X^2^ = 4.93, *p* = 0.026) were more common in the NFAI group as compared with the control group. There was no statistically significant difference in the incidence of MS in the study group compared to the control. Such differences were also not demonstrated in the incidence of abdominal obesity or dyslideaemia (Table 5). Antihypertensive treatment received 27 people from NFAI group and 11 from control group, lipid lowering drug therapy received 18 people from NFAI group and 7 participants of controls.

In all 7 patients with NFAI, metabolic syndrome was diagnosed based on abdominal obesity, hypertension and IFG. In addition, one person had a low HDL-C level.

In the NFAI group statistically significant positive correlations between CIMT and age (r = 0.1872, *p* = 0.0031), waist circumference (r = 0.316, *p* = 0.0286), diastolic blood pressure (r = 0.3382, *p* = 0.0187) were revealed. No correlation was found between CIMT and systolic blood pressure, fasting blood glucose and glucose in 2 h OGTT, lipid levels or smoking (Table 6).

Hormonal assessment of hypophysis-adrenal axis in AI group showed mean 8 a.m. cortisol 13.4 ± 3.41 μg/dL, 10 p.m. cortisol 3.6 ± 1.66 μg/dL, cortisol in LDDST 1.23 ± 0.41 ug/dL, morning ACTH 10.32 ± 7.36 pg/mL and DHEAS level 115.83 ± 92.13 μg/dl. ARR calculated as PAC (ng/dL) divided by PRA (ng/mL/h) was 8.44 ± 3.81, androstendione 1.08 ± 0.65 ng/mL, plasma free metanephrines level 0.842 +/− 0.062 nmol/L, urinary metanephrine 468.62 ± 183.6 μg/day and urinary normetanephrine 434.2 ± 163.5 μg/day.

The analysis of associations between CIMT and values of steroidogenesis hormones showed a statistically significant positive correlation between CIMT and serum cortisol levels after suppression with 1 mg dexamethasone (r = 0.33, *p* = 0.02). There was no correlation between CIMT and morning cortisol and ACTH levels, cortisol levels at 10 p.m., or DHEA-S levels (Table 7).

## 4. Discussion

A higher prevalence of abdominal obesity, hypertension, as well as carbohydrate and lipid metabolism disorders has been found in patients with AIs [8,21,22,23,24,25,26,27,28,29]. Each of these disorders contributing to metabolic syndrome is considered an independent risk factor for cardiovascular disease occurrence and cardiovascular disease- related death. Yet, when they co-occur, the total cardiovascular risk is significantly higher than when individual components appear separately [30].

Our study did not reveal significant differences in the prevalence of abdominal obesity in the analysed groups. However, despite the fact that both of the groups were matched for BMI, waist circumference in the NFAI group was significantly higher than in the control group (97.6 ± 14 vs. 89.1 ± 9.5, respectively; *p* < 0.01). Similar findings revealed also studies of other authors [8,10,31]. To date, the relationship between excessive body weight and AIs has been explained as a possible consequences of minimal hormonal secretion not detectable by current diagnostic methods. However, the observed anabolic and mitogenic effects of insulin on the adrenal cortex have led to the hypothesis suggesting a potential two-way relationship between obesity and AIs [10,32]. It should be noted that adipose tissue, especially visceral fat, is regarded as an important component of the endocrine system. It releases adipokines, and thus, it plays a significant role in the regulation of glucose and lipid metabolism, leading to disorders observed in individuals with AIs [29,33,34].

In a study by Garrapa et al. the glucose area under the curve after an OGTT was significantly larger in patients with Cushing’s syndrome and NFAI as compared to the control group [21]. These alterations suggest that a subtle cortisol hypersecretion is probably present in AIs and it may be the factor promoting alterations of body composition and metabolic parameters. Reincke et al. identified IR and abdominal obesity in all the 13 assessed patients with NFAI [24]. Similar conclusions were reached by Peppa et al. in their study including 29 patients with NFAI. HOMA-IR was statistically significantly higher in the NFAI group compared to the control group (3.6 ± 1.7 vs. 2.4 ± 1.5, respectively; *p* = 0.009) [28]. It is likely that because of the insufficient number of participants in the assessed groups, our study did not demonstrate a higher HOMA-IR value in the NFAI group compared to the control group. The fasting insulin level in the NFAI group was statistically significantly higher compared to the control group (11.4 ± 4.9 vs. 8.9 ± 5.8, *p* = 0.03). Importantly, the correlation between fasting insulin levels and cortisol levels in the 1 mg dexamethasone test was close to reaching statistical significance (r = 0.254; *p* = 0.08).

IR and IGT are known to be among the major metabolic complications of glucocorticoid (GC) excess, which can ultimately lead to the development of type 2 DM [35,36]. GCs promote hepatic gluconeogenesis, inhibit the secretion and action of insulin, promote differentiation and proliferation of adipocytes, redistribution of fat, and decrease lipoprotein-lipase activity [9]. It has been noted that fasting glucose testing has a relatively low sensitivity when it comes to detecting abnormal carbohydrate metabolism in people with endogenous hypercortisolaemia [36]. In patients with Cushing’s syndrome, abnormal carbohydrate metabolism is observed in up to 43–84% [37,38,39]. Previous studies indicate that carbohydrate tolerance disorders are also more common in the NFAI group, although their fasting glucose levels remain normal [36]. In our study, the NFAI group showed significantly higher 2 h OGTT glucose levels, significantly more frequent IGT compared to the control group (27% vs. 0.7%, respectively; *p* < 0.01). Terzolo et al. also demonstrated that the 2-h post-challenge glucose level was significantly higher in the NFAI group than in the control group [31]. Such results make one suspect that seemingly benign NFAIs may temporarily secrete supraphysiological amounts of GCs. Although there was no correlation between cortisol levels in the LDDST and glucose levels in the 2 h OGTT, the relationship was close to reaching statistical significance (r = 0.263, *p* = 0.077). On the other hand, a less frequent occurrence of IFG was observed in the NFAI group than in the control group (20.8% vs. 47.7%, respectively; *p* < 0.01). This may be due to the fact that DM2 was more frequently diagnosed in the NFAI group based on blood glucose in the 2 h OGTT. As such patients met the exclusion criterion established for the present study, they were not considered in the statistical analyses.

The prevalence of hypertension in the general adult population is app. 31% and increases with age. The percentage is greater than 50% in the 60-69 age group and app. 75% in those over 70 years of age [40]. In our study, the mean age of the NFAI group was 58 ± 8 years, and the prevalence of hypertension among patients in that group was significantly higher than in the control group (56% vs. 27%, respectively; *p* < 0.01). It was also higher than that determined on the basis of available cohort studies. Higher systolic and diastolic blood pressure values were observed in the NFAI group, and hypertension was more common there than in the control group. Our data are consistent with the findings of other authors [22,23,29,31]. In the study by Rabelo et al. patients with AI presented higher frequency of hypertension (74.1 vs. 57.8%; *p* = 0.02), resistant hypertension (45.4 vs. 9.4%; *p* < 0.001) compared to the controls, respectively. NFAI and ACS patients presented similar frequency of arterial hypertension (70.8 vs. 79.3%) and resistant hypertension (41.3 vs. 51.1%) [41].

The long-term effects of mild autonomous cortisol production may be harmful and surgical treatment could be a considered option. There are reports suggesting that it is possible to improve metabolic parameters in patients with NFAI after they undergo adrenalectomy. In such patients weight loss, lowered blood pressure, as well as lowered lipid and fasting glucose levels have been observed after surgery [42,43]. It may suggest that a subtle cortisol hypersecretion is probably present in NFAI patients and it may be the factor promoting alterations of metabolic profile and body composition. In the light of current recommendations adrenalectomy in NFAI patients is still controversial and requires further research.

In the present study, the NFAI group showed a significantly higher CIMT value compared to the control group (*p* < 0.01). There was a positive correlation between the CIMT and age (r = 0.373, *p* < 0.01), diastolic blood pressure (r = 0.338, *p* = 0.19) and waist circumference (r = 0.316, *p* = 0.029). Importantly, a positive correlation was also observed between the CIMT value and the LDDST cortisol level (r = 0.333, *p* = 0.02), which indicates that AIs may in fact produce small amounts of GCs that, in consequence, may cause morphological and functional changes in blood vessels. Our results are consistent with the observations of other authors [11,12,44,45]. Androulakis et al. presented significantly higher CIMT values in cortisol secreting AIs (CSAIs) compared to the NFAI group and the control group. Patients with NFAI had higher CIMT values compared to the control group. The study determined a positive correlation between CIMT values and cortisol levels in the LDDST. Authors suggested that increased prevalence of CV risk factors described in NFAI patients could result from either a mild cortisol excess that cannot be detected with available diagnostic methods or its periodic secretion. [7] In a study by Emral et al., the CIMT had a positive correlation with age (r = 0.245, *p* = 0.004), HOMA-IR (r = 0.490, *p* < 0.001), fasting insulin concentration (r = 0.432, *p* < 0.001), and TC concentration (r = 0.267, *p* = 0.002) [12]. Similar conclusions were drawn by Cansu et al., based on a study of 35 patients with NFAI without traditional CVD risk factors such as DM2, HT or hyperlipidaemia. The study showed a positive correlation of the CIMT with age (r = 0.484, *p* < 0.005), triglycerides (r = 0.378, *p* < 0.005) and cortisol levels after the LDDST (r = 0.346, *p* < 0.005). Authors speculated that increased level of CIMT may be attributable to subtle and undetectable cortisol autonomy in NFAI patients [13].

NFAI is diagnosed on the basis of hormonal tests (normal diurnal cortisol rhythm, ACTH and DHEAS levels, cortisol levels dl in LDDST below 1.8 µg/dL), which seem to be contradicted by the results of biochemical tests and numerous metabolic disorders in this group of patients. Some authors state that there is a slight excess of cortisol in patients with NFAI that cannot be detected by existing diagnostic tests or due to intermittent hormonal secretion. This raises suspicion that LDDST as a recommended tool for diagnosis subtle autonomic cortisol secretion is not perfect. According to the results of our research, normal hormonal tests may occur in patients who already have metabolic and vascular complications. For this reason, further research is needed of patients with apparently non-functional adenomas.

## 5. Limitations

It is a limitation of the present study that the NFAI group which underwent analysis was relatively small. Additionally, no hormonal tests were performed in the control group. However, exposing patients without known adrenal lesions to additional hormonal tests was assumed to be unreasonable, especially if such tests were to be associated with taking a suppressive dose of GCs. This is a cross-sectional study and thus it is not possible to determine a causal relationship between AI and the variables observed.

## 6. Conclusions

NFAIs are associated with the occurrence of metabolic disorders and early vascular changes in the form of increased CIMT values. The undertaken study indicates that NFAIs with normal cortisol inhibition in LDDST test may have adverse metabolic and vascular effects. Patients with AI presenting with hypertension, impaired carbohydrate metabolism, dyslipidemia should be considered for primary and secondary prevention of CVD. The underlying mechanisms of these findings are unknown, hence further studies are needed.

## Figures and Tables

**Table 1:** Exclusion criteria.

Exclusion criteria: -age below 18 years or over 69 years -hormonally active adrenal adenoma -diabetes mellitus -chronic kidney disease (stage 3a renal failure with eGFR < 60 mL/min) -ischaemic heart disease -cardiac insufficiency -uncompensated hypertension -cerebrovascular disease -peripheral arterial disease -acute and chronic inflammatory diseases -malignant neoplasm -suspected or diagnosed mental disorder -use of medications that may affect the hypothalamic-pituitary-adrenal axis -pregnancy

**Table 2:** Characteristics of the analysed groups. * Mann-Whitney U test.

Parameters	NFAI Group (48)	Control Group (44)	*p*-Value
Women/Men (*n*)	32/16	29/15	
Age (years)	58.6 ± 9	57 ± 7	0.34
BMI (kg/m^2^)	28.7 ± 4.6	26.8 ± 4.3	0.69
Waist circumference (cm)	97.6 ± 14	89.1 ± 9.5	<0.01 *
Systolic pressure (mmHg)	131.7 ± 15.1	123.6 ± 10.8	<0.01 *
Diastolic pressure (mmHg)	82.5 ± 9.9	75.7 ± 9.2	<0.01 *
Heart rate (per minute)	77.2 ± 11	72.5 ± 8.9	0.176

BMI-Body mass index.

**Table 3:** Laboratory data and intima-media thickness. * Mann-Whitney U test.

Parameters	NFAI Group (*n* = 48)	Control Group (*n* = 44)	*p*-Value
Fasting blood glucose (mg/dL)	96.1 ± 12	100.4 ± 11	0.7
Blood glucose in 2 h OGTT (mg/dL)	127.7 ± 38.2	105.1 ± 27.9	0.04
Fasting insulin uU/mL	11.4 ± 4.9	8.9 ± 5.8	0.03
HOMA-IR Index	2.72 ± 1.23	2.26 ± 1.64	0.13
Total cholesterol (mg/dL)	208.7 ± 43.1	207.4 ± 31.8	0.94
LDL cholesterol (mg/dL)	139.6 ±44.8	139.68 ± 31.01	0.94 *
HDL cholesterol (mg/dL)	63.18 ± 15.12	63.61 ± 17.76	0.82 *
Non-HDL cholesterol (mg/dL)	144.8 ± 44.41	143.75 ± 29.63	0.91 *
Triglycerides (mg/dL)	101.72 ± 45.2	109.63 ± 48.7	0.62
Creatinine (mg/dL)	0.81 ± 0.14	0.85 ± 0.15	0.79
eGFR (mL/min)	82.87 ± 18.65	79.84 ± 12.86	0.015
Sodium (mmol/L)	138.54 ± 18.01	139.93 ± 1.99	0.02 *
Potassium (mmol/L)	4.29 ± 0.34	4.38 ± 0.34	0.2 *
CIMT (mm)	0.64 ± 0.17	0.47 ± 0.11	<0.01 *

CIMT-Carotid Intima-Media Thickness; eGFR-estimated glomerular filtration rate; HDL-C–high-density lipoprotein cholesterol; HOMA-IR-Homeostasis Model Assessment-Insulin Resistance; LDL-C–low-density lipoprotein cholesterol; Non-HDL cholesterol-non-high-density lipoprotein cholesterol; OGTT-Oral glucose tolerance test; TC-total cholesterol.

**Table 4:** Prevalence of conditions that have effect on vascular remodeling.

Parameters	NFAI Group (*n* = 48)	Control Group (*n* = 44)	*p*-Value
Hypertension	27 (56%)	12 (27%)	<0.01
IFG	10 (20.8%)	21 (47.7%)	<0.01
IGT	13 (27%)	4 (0.9%)	0.026
Dyslipidaemia	42 (85.7%)	42 (87.5%)	0.11
Smoker	9 (19%)	9 (20.5%)	0.8
Metabolic syndrome	7 (14%)	10 (22.7%)	0.31

IFG-impaired fasting glucose; IGT-impaired glucose tolerance.

**Table 5:** Incidence of components of MS diagnosis (IDF 2009).

Parameters	Study Group (*n* = 48)	Control Group (*n* = 44)	*p*-Value
Central obesity	41 (85%)	32 (72.7%)	0.501
Hipertriglicerydemia	4 (0.08%)	5 (11.4%)	0.625
Low HDL-cholesterol	1 (0.02%)	4 (0.1%)	0.139
Hypertension	27 (56%)	12 (27%)	<0.01
IFG	10 (20.8%)	21 (47.7%)	<0.01

IFG- impaired fasting glucose; HDL cholesterol- high-density lipoprotein cholesterol.

**Table 6:** Correlation between clinical and biochemical parameters and CIMT in patients with NFAI.

Parameters	CIMT	
	r	*p*
Age	0.373	<0.01
BMI	0.201	0.171
Waist circumference	0.316	0.029
Systolic blood pressure	0.271	0.063
Diastolic blood pressure	0.338	0.019
Fasting glucose	0.035	0.815
Fasting insulin	0.122	0.409
HOMA	0.091	0.538
Glucose in 2 h OGTT	−0.005	0.976
Total cholesterol	0.039	0.793
LDL-C	0.022	0.88
HDL-C	0.273	0.060
Non-HDL-C	−0.839	0.575
TG	−0.051	0.733
Sodium	0.218	0.138
Potassium	0.237	0.105

CIMT-Carotid Intima-Media Thickness; HDL-C–high-density lipoprotein cholesterol; HOMA-IR- Homeostasis Model Assessment-Insulin Resistance; LDL-C–low-density lipoprotein cholesterol; Non-HDL cholesterol-non-high-density lipoprotein cholesterol; OGTT-Oral glucose tolerance test; TC-total cholesterol.

**Table 7:** Correlation between hormonal parameters and CIMT in patients with NFAI.

Parameters	CIMT	
	r	*p*
Cortisol 8:00 a.m.	0.1607	0.2753
Cortisol 10:00 p.m.	0.2238	0.1262
ACTH 8:00 a.m.	0.1691	0.2504
Cortisol after LDDST	0.3334	0.0205
DHEA-S	−0.1587	0.2813

ACTH-adrenocorticotropic hormone; DHEAS-dehydroepiandrosterone; LDDST-low-dose dexamethason supression test.

## Data Availability

Not applicable.
